# Pyrotinib combined with trastuzumab and chemotherapy for the treatment of human epidermal growth factor receptor 2-positive metastatic breast cancer: a single-arm exploratory phase II trial

**DOI:** 10.1007/s10549-022-06770-6

**Published:** 2022-10-30

**Authors:** Xiao-Feng Xie, Qiu-Yi Zhang, Jia-Yi Huang, Li-Ping Chen, Xiao-Feng Lan, Xue Bai, Lin Song, Shui-Ling Xiong, Si-Jia Guo, Cai-Wen Du

**Affiliations:** 1 Present Address: Department of Medical Oncology, National Cancer Center/ National Clinical Research Center for Cancer/Cancer Hospital & Shenzhen Hospital, Chinese Academy of Medical Sciences and Peking Union Medical College, No. 113 Baohe Road, Longgang District, Shenzhen, 518116 Guangdong People’s Republic of China; 2 School of Pharmaceutical Science, Shenzhen, Health Science Center, Shenzhen University, No. 3688, Nanhai Road, Nanshan District, Shenzhen, 518060 Guangdong People’s Republic of China

**Keywords:** Pyrotinib, Trastuzumab, Chemotherapy, Human epidermal growth factor receptor 2 (HER2), Metastatic breast cancer (MBC)

## Abstract

**Purpose:**

A substantial need for effective and safe treatment options is still unmet for patients with heavily pre-treated human epidermal growth factor receptor 2 (HER2)-positive metastatic breast cancer (MBC). Herein, we assessed the efficacy and safety of pyrotinib plus trastuzumab and chemotherapy in patients with heavily treated HER2-positive MBC.

**Methods:**

In this single-arm exploratory phase II trial, patients with HER2-positive MBC previously treated with trastuzumab plus lapatinib or pertuzumab, received pyrotinib plus trastuzumab and chemotherapy. The primary end point was progression-free survival (PFS) in the total population (TP). Secondary end points included PFS in the subgroup with brain metastases (Sub-BrM), confirmed objective response rate (ORR), clinical benefit rate (CBR), disease control rate (DCR), exploration of predictive factors of PFS, and safety.

**Results:**

Between November 1, 2018, and March 31, 2021, 40 patients were eligible for this study. The median PFS reached 7.5 months (95% confidence interval [CI] 4.7 to 9.9 months) and 9.4 months (95% CI 6.6 to 12.1 months) in the TP and Sub-BrM, respectively. ORR was 50.5% (20/40). CBR was 75.5% (30/40) and DCR reached 97.5% (39/40). Cox univariate and multivariate analyses demonstrated that liver or/and lung metastases was the significant adverse prognostic factor for PFS (*p* = 0.018; *p* = 0.026; respectively). The most frequent grade 3 or 4 treatment-related adverse events were diarrhea, neutropenia and leukopenia. No new safety signals were observed.

**Conclusion:**

Pyrotinib plus trastuzumab and chemotherapy offered a promising option with manageable safety profile for heavily pre-treated HER2-positive MBC, especially for those without liver or/and lung metastases.

## Introduction

Breast cancer (BC) has surpassed lung cancer as the most commonly diagnosed malignancy in women according to Global Cancer statistics 2020 [1]. Approximately 15 to 20% of BC cases exhibit overexpression of the human epidermal growth factor receptor 2 (HER2) or/and amplification of the coding gene *ERBB2*, which is defined as HER2-positive subtype [2]. HER2-positive BC is more aggressive and prone to recurrence than HER2-negative tumors [3]. In the last decades, the clinical outcome of HER2-positive BC has been significantly improved since the introduction of HER2-targeted drugs mainly including monoclonal antibodies, tyrosine kinase inhibitors (TKIs) and antibody drug conjugates (ADCs). Currently, the standard of care for HER2-positive metastatic breast cancer (MBC) involves trastuzumab plus pertuzumab and a taxane as the first-line regimen, followed by second-line trastuzumab emtansine (T-DM1) or fam-trastuzumab deruxtecan-nxki (ds8201) for patients whose disease has progressed on prior dual HER2 blockade [4]. However, a large proportion of patients with HER2-positive MBC fail to be treated with standard second-line regimens due to economic and drug accessibility factors. Moreover, most patients inevitably experience disease progression on anti-HER2-targeted therapy due to de novo or acquired resistance. Therefore, it is necessary to explore novel approaches to overcome drug resistance. Key strategies include efficient suppression of the HER2 signaling pathway by dual blockade and development of more effective anti-HER2 therapies like antibody–drug conjugates, new anti-HER2 antibodies, bispecific antibodies and novel TKIs [5].

Brain metastases (BrM) is a common complication of advanced malignant disease and occurs in 1/3 of HER2-positive metastatic breast cancer [6]. Central nervous system-directed local therapies, including surgical resection and radiotherapy, are the foundations of BrM management [7]. Besides, TKIs and several chemotherapeutic drugs with blood–brain barrier penetrability have demonstrated efficacy in HER2-positive MBC patients with BrM. Despite sequential local and systemic treatment, resistance inevitably develops and options are limited for the control of BrM.

Studies have confirmed the activity of continued trastuzumab treatment beyond progression (TBP). The subgroup analysis of observational HERMINE study suggested that trastuzumab TBP offered a survival benefit to MBC patients treated with first-line trastuzumab [8]. Furthermore, a phase III study in HER2-positive BC showed that patients receiving continued trastuzumab TBP had a better post-progression survival than those not receiving (18.8 m *vs* 13.3 m, *p* = 0.02) [9]. Meanwhile, the potent anti-tumor efficacy of TKIs including lapatinib, neratinib, tucatinib and pyrotinib has been demonstrated in several pivotal studies [10–14]. Pyrotinib is an orally administered irreversible pan-ErbB TKI which shows anti-tumor activity and acceptable safety profile in HER2-positive advanced and metastatic breast cancer [14]. In a phase II study, pyrotinib plus capecitabine yielded statistically significant better overall response rate (ORR) and progression-free survival (PFS) than lapatinib plus capecitabine in patients with HER2-positive MBC previously treated with taxanes, anthracyclines, and/or trastuzumab [15]. Based on this phase II study, pyrotinib in combination with capecitabine received its first conditional approval in China for the treatment of HER2-positive, advanced or metastatic breast cancer previously exposed to anthracycline or taxane chemotherapy. Afterwards, a multi-center, open-label, randomized, controlled, phase III trial (PHOEBE) confirmed the efficacy and safety of pyrotinib plus capecitabine in patients with disease progression on previous trastuzumab [16]. Another phase III trial PHENIX further verified that pyrotinib plus capecitabine significantly improved PFS and ORR compared with capecitabine monotherapy in trastuzumab-treated patients with HER2-postive advanced or metastatic breast cancer, including those with brain metastases [17].

Dual HER2 blockade by trastuzumab plus TKI, simultaneously targeting the extra- and intra-cellular domains of HER2, showed encouraging anti-tumor activity in BC, including early breast cancer and MBC with intracranial metastases [18–21]. Our study aimed to explore the activity and safety of pyrotinib combined with trastuzumab and chemotherapy of physician’s choice in heavily pre-treated patients with HER2-positive MBC with or without BrM.

## Methods

### Patients and data collection

From November 1, 2018, to March 31, 2021, female patients aged ≥ 18 years old with histologically confirmed HER2-positive MBC at National Clinical Research Center for Cancer/Cancer Hospital & Shenzhen Hospital (Shenzhen, Guangdong, China) were enrolled in this study. HER2 status was determined by central review based on immunohistochemistry (IHC) or fluorescence in situ hybridization (FISH) examination using primary or metastatic lesion samples. Patients had been previously treated with trastuzumab, pertuzumab and/or lapatinib in the (neo)adjuvant/metastatic setting. Additional requirements included an Eastern Cooperative Oncology Group (ECOG) performance status score of 0 or 1 (on a 5-point scale by which greater scores reflect higher degree of disability); measurable lesions; and adequate organ function. Patients were excluded if they had previously received treatment with pyrotinib for metastatic disease; had symptomatic brain metastasis which necessitates immediate local intervention; or had leptomeningeal disease. All procedures performed involving human participants were in accordance with the 1964 Helsinki Declaration and its later amendments or comparable ethical standards. This study was also approved by the institutional review board and ethics committee. All patients provided written informed consent for use of the medical information for research purpose.

### Treatment

All eligible patients were treated with pyrotinib (240–400 mg orally once daily), trastuzumab (6 mg per kilogram of body weight intravenously per 21 days, with an initial loading dose of 8 mg per kilogram) and single chemotherapeutic agent (nab-paclitaxel, 260 mg per square meter of body-surface area intravenously on day 1 of each 21-day cycle; or capecitabine, 1000 mg per square meter of body-surface area orally twice daily on days 1 to 14 of each 21-day cycle; or gemcitabine, 1000 mg per square meter of body-surface area intravenously on days 1 and 8 of each 21-day cycle; or vinorelbine, 60 mg per square meter of body-surface area orally on days 1 and 8 of each 21-day cycle and dose elevation to 80 mg per square meter of body-surface area was allowed if well tolerated).

### Follow-up and assessment

All patients were followed up until January 20, 2022. Disease response was evaluated according to imaging reports from serial clinical assessments. Patient/disease response assessments were performed at baseline, every 6 weeks for 24 weeks, and every 9 weeks thereafter, including performance status, history, laboratory examinations, electrocardiogram**,** contrast-enhanced spiral computed tomography (CT) or positron emission tomography/CT (PET/CT), and contrast-enhanced magnetic resonance imaging (MRI). MRI of the head was obtained for all patients at baseline and head scans were repeated at the frequency described above if BrM had been detected at baseline. MRI of the breast was not required. Disease was evaluated in accordance with Response Evaluation Criteria in Solid Tumors (RECIST) criteria, version 1.1 [22]. Safety was assessed mainly via the incidence of treatment-related adverse events (TRAEs) using the National Cancer Institute Common Terminology Criteria for Adverse Events version 5.0.

### End points

The primary end point was PFS (calculated from the date of first treatment with double targeted therapy to the date of documented disease progression or death from any cause or the last follow-up visit) in TP. The secondary end points included PFS in the subgroup with BrM (Sub-BrM) at baseline; objective response rate (ORR, defined as the percentage of patients who had a confirmed complete response or partial response); clinical benefit rate (CBR, defined as the percentage of patients who had a confirmed complete response or partial response or stable disease for at least 24 weeks); disease control rate (DCR, defined as the percentage of patients who had a confirmed complete response or partial response or stable disease for at least 4 weeks); exploration of predictive factors of PFS; and safety.

### Statistical analysis

All data were analyzed using SPSS 23.0 statistical software (SPSS, Inc., Chicago, IL, USA) and GraphPad Prism 5 software (GraphPad Software, Inc., La Jolla, CA, USA). Descriptive analysis was utilized to display clinicopathological features. The Kaplan–Meier method was used to estimate PFS and 95% confidence intervals (CIs) for the total population and the subgroups. Cox univariate and multivariate models were used to determine the predictive value of variables for PFS. In the analysis of progression-free survival, data from patients who did not have any documented event, were lost to follow-up or died from any cause were censored at the last date when the patient was known to be event-free. All reported *p* values were two-sided, with *p* < 0.05 being regarded as statistically significant.

## Results

### Patient clinicopathological characteristics

Between November 1, 2018, and March 31, 2021, a total of 40 patients were eligible for this study and included in the final analysis. In TP, the median age at diagnosis was 46 years (range 31–62 years). 33 patients (82.5%) were aged over 35 years old at diagnosis. ECOG performance status was 0 in 21 patients (52.5%) and 1 in 19 patients (47.5%). For HER2 status, HER2 3+ by IHC was recorded in 32 patients (80.0%), and HER2 2+ by IHC and amplification by FISH in 8 patients (20.0%). 21 patients (52.5%) were estrogen receptor (ER) and/or progesterone receptor (PR) positive and 19 patients (47.5%) were ER and PR negative. 39 patients (97.5%) had been treated with trastuzumab in the (neo)adjuvant or metastatic setting; 6 patients had received pertuzumab and trastuzumab as first-line therapeutic regimen; 21 patients (52.5%) received anti-HER2 therapy with TKI (lapatinib) for their metastatic disease; none of them was previously treated with TDM-1. 15 patients (37.5%) had brain metastases and 27 patients (67.5%) had lung or/and liver metastases at baseline. Of those patients with brain metastases, 7 patients had received central nervous system (CNS) local therapies including whole-brain radiotherapy (WBRT), stereotactic radiotherapy (SRT) and surgery plus SRT before study treatment; 3 patients whose intracranial disease progressed while remission of extra-cranial lesions lasted, received SRT with unchanged systemic therapy. 13 patients (32.5%) received at least 3 lines of therapy in the metastatic setting. Combined chemotherapeutic agents included vinorelbine (25, 62.5%), capecitabine (6, 15.0%), nab-paclitaxel (5, 12.5%) and gemcitabine (4, 10.0%). Patient clinicopathological characteristics at baseline were shown in Table 1.

**Table 1:** Patient clinicopathological characteristics at baseline

Characteristics	Cases (%)
ECOG	
0	21 (52.5)
1	19 (47.5)
Age at diagnosis	
≤ 35 years	7 (17.5)
> 35 years	33 (82.5)
Location	
Left	27 (67.5)
Right	13 (32.5)
HER2 status	
IHC 3+	32 (80.0)
IHC 2+ and FISH+	8 (20.0)
Hormone-receptor status	
ER and/or PR positive	21 (52.5)
E R and PR negative	19 (47.5)
Previous anti-HER2 antibody treatment in metastatic setting	
Yes	37 (92.5)
No	3 (7.5)
Previous anti-HER2 TKI treatment	
Yes	21 (52.5)
No	19 (47.5)
Brain metastases	
Yes	15 (37.5)
No	25 (62.5)
Liver or/and lung metastases	
Yes	27 (67.5)
No	13 (32.5)
Number of previous treatment lines	
≤ 2	27 (67.5)
> 2	13 (32.5)
Combined chemotherapeutic drug	
Vinorelbine	25 (62.5)
Capecitabine	6 (15.0)
Nab-paclitaxel	5 (12.5)
Gemcitabine	4 (10.0)

*ECOG* Eastern Cooperative Oncology Group, *HER2* human epidermal growth factor receptor 2, *IHC* immunohistochemistry, *ER* estrogen receptor, *PR* progesterone receptor

### Efficacy

**Fig. 1:**
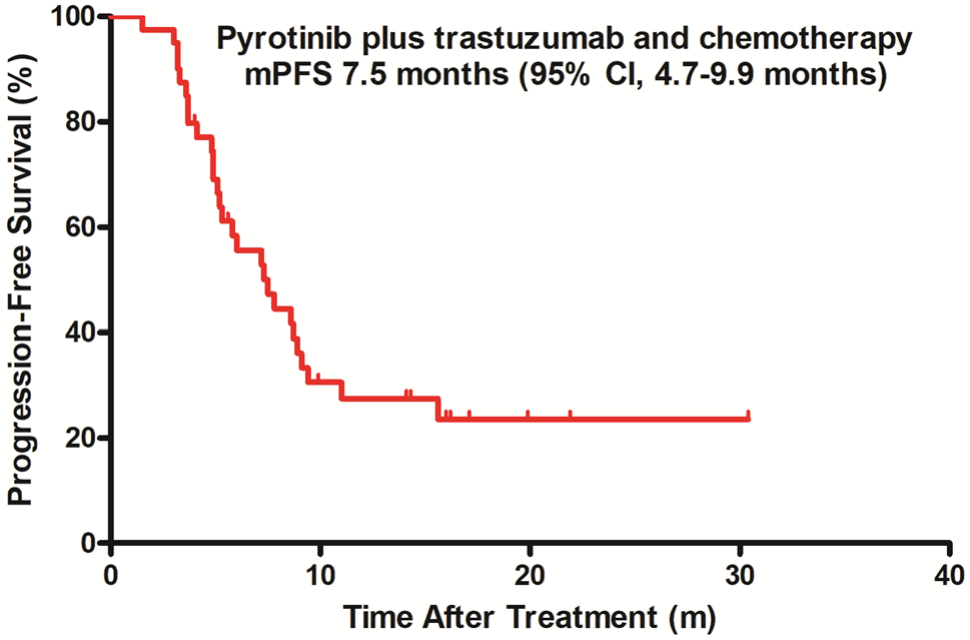
Kaplan–Meier estimates of progression-free survival in the total population. *mPFS* median progression-free survival, *CI* confidence interval

**Fig. 2:**
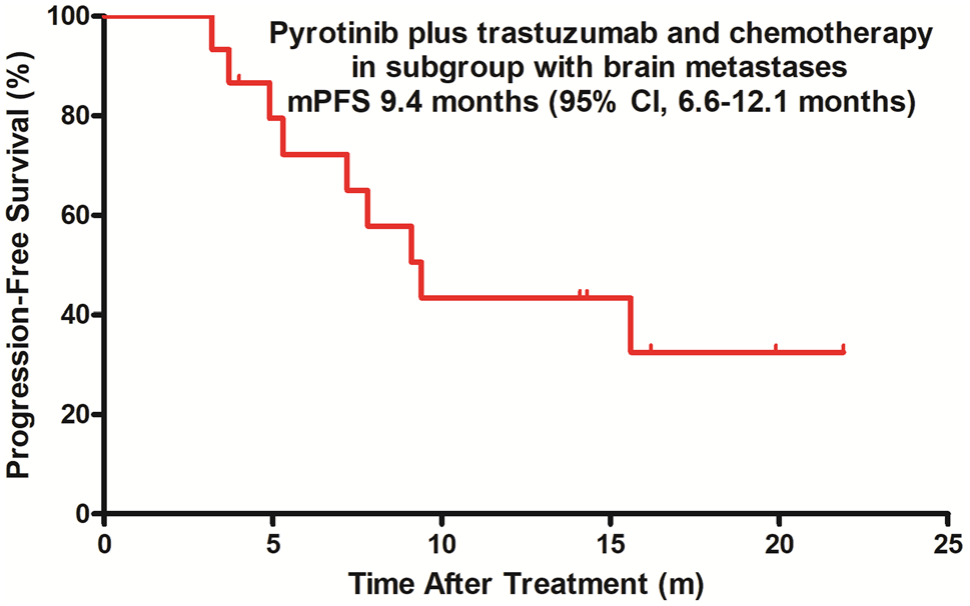
Kaplan–Meier estimates of progression-free survival in the subgroup with brain metastases. *mPFS* median progression-free survival, *CI* confidence interval

By the end of follow-up, 32 patients had experienced disease progression and 3 patients had died. The median duration of PFS was 7.5 months in TP (95% CI 4.7 to 9.9 months, Fig. 1) and was 9.4 months in Sub-BrM (95% CI 6.6 to 12.1 months, Fig. 2). Objective response was seen in 20 of 40 patients (including 1 patient with complete response and 19 patients with partial response, 50.0%, Table 2). All patients with objective response achieved disease remission within four cycles (12 weeks) after treatment administered. Clinical benefit was observed in 30 of 40 patients (75.5%) and disease control in 39 of 40 patients (97.5%, Table 2).

**Table 2:** Efficacy outcomes in the total population and subgroup with brain metastases at baseline

End points	
Median progression-free survival, months (95% CI)	7.5 (4.4 to 7.9)
Median progression-free survival in subgroup with brain metastases, months (95% CI)	9.4 (6.6 to 12.1)
Type of response, no. (%)	
Complete	1 (2.5)
Partial	19 (45.0)
Stable disease	19 (50.0)
Disease progression	1 (2.5)
Objective response rate, no. (%)*	20 (50.0)
Clinical benefit rate, no. (%)**	30 (75.5)
Disease control rate, no. (%)***	39 (97.5)

Abbreviations: CI confidence interval

*Include complete and partial response

**Include complete and partial response and stable disease lasting for at least 24 weeks

***Include complete and partial response and stable disease lasting for at least 4 weeks

**Fig. 3:**
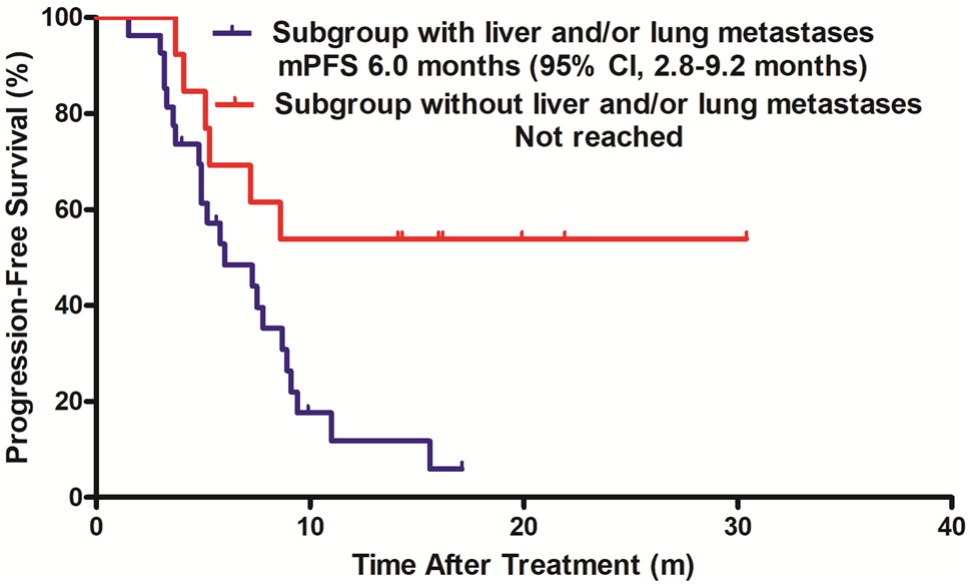
Kaplan–Meier estimates of progression-free survival in subgroups with or without liver and/or lung metastases. *mPFS* median progression-free survival, *CI* confidence interval

Univariate and multivariate analyses were performed to identify predictive factors of PFS for the 40 patients. By means of Cox univariate and multivariate analyses, we found that liver or/and lung metastases at baseline was the significant adverse prognostic factor for PFS (hazard ratio [HR] 0.322; 95% CI 0.132 to 0.831; *p* = 0.018; HR 0.281; 95% CI 0.093 to 0.876; *p* = 0.026; respectively; Table 3). The median PFS was 6.0 months (95% CI 2.8 to 9.2 months) in the subgroup with liver or/and lung metastases and was not reached in the subgroup without (Fig. 3).

**Table 3:** Cox regression analysis for progression-free survival (*N* = 40)

Variables	Univariate analysis			Multivariate analysis		
	HR	95% CI	*p* value	HR	95% CI	*p* value
Age at diagnosis, years (≤ 60 vs > 60)	1.277	0.484–3.368	0.622			
Location (left vs right)	1.219	0.547–2.716	0.629			
HER2 status (IHC 3+ vs IHC2+ and FISH+)	1.017	0.355–2.687	0.972			
Hormone-receptor status (ER and/or PR positive vs ER and PR negative)	0.639	0.299–1.367	0.248			
Previous anti-HER2 antibody treatment in metastatic setting (yes vs no)	0.947	0.225–4.037	0.953			
Previous anti-HER2 TKI treatment (yes vs no)	0.958	0.451–2.033	0.911			
Brain metastases (yes vs no)	1.877	0.843–4.179	0.123			
Liver or/and lung metastases (yes vs no)	0.322	0.132–0.831	0.018	0.281	0.093–0.876	0.026
Number of previous treatment lines (≤ 2 vs > 2)	1.190	0.548–2.585	0.660			
Combined chemotherapeutic drug (Vinorelbine vs Cepecitabine vs Nab-paclitaxel)	1.197	0.834–1.717	0.329			

*HR* hazard ratio, *CI* confidence interval, *HER2* human epidermal growth factor receptor, *IHC* immunohistochemistry, *FISH* fluorescence in situ hybridization, *ER* estrogen receptor, *PR* progesterone receptor, *TKI* tyrosine kinase inhibitor

### Safety

All treatment-related adverse events (TRAEs) reported in the study were summarized in Table 4. The most common adverse events (frequency ≥ 10%) were diarrhoea (85.0%), leukopenia (42.5%), neutropenia (37.5%), fatigue (37.5%), vomiting (32.5%), and nausea (12.5%) in turn. Other recorded adverse events included hand-foot syndrome (10.0%), aspartate aminotransferase increase (10.0%), anorexia (7.5%), alanine aminotransferase increase (5.0%), anemia (5.0%), peripheral neurotoxicity (5.0%), rash (2.5%) and thrombocytopenia (2.5%). The most frequent grade 3 to 4 adverse events were diarrhoea (25.0%), neutropenia (15.0%), and leukopenia (12.5%). Grade 4 adverse events occurred in 1 patient (leukopenia and febrile neutropenia). No grade 4 diarrhea or cardiac-related events were reported. No one discontinued study treatment or died owing to TRAEs. However, 14 patients (35.0%) experienced pyrotinib dose reduction because of diarrhoea (10 patients reduced from 400 to 320 mg and 4 patients from 400 to 240 mg). 4 patients experienced chemotherapeutic drug dose reduction (nab-paclitaxel in 1 patient, capecitabine in 1 patient, and vinorelbine in 2 patients). Diarrhoea, the most common adverse event, mostly occurred within the first two weeks after treatment initiation and could be managed by the administration of loperamide or/and pyrotinib dose reduction.

**Table 4:** Treatment-related adverse events in total population

Adverse events	Any grade (%)	Grade ≥ 3 (%)
Diarrhoea	34 (85.0)	10 (25.0)
Leukopenia	17 (42.5)	5 (12.5)
Neutropenia	15 (37.5)	6 (15.0)
Fatigue	15 (37.5)	0
Vomiting	13 (32.5)	1 (2.5)
Nausea	5 (12.5)	0
Hand-foot syndrome	4 (10.0)	0
Aspartate aminotransferase increase	4 (10.0)	1 (2.5)
Anorexia	3 (7.5)	0
Alanine aminotransferase increase	2 (5.0)	0
Anemia	2 (5.0)	0
Peripheral neurotoxicity	2 (5.0)	0
Rash	1 (2.5)	0
Thrombocytopenia	1 (2.5)	1 (2.5)

## Discussion

In this single-center study, pyrotinib combined with trastuzumab and chemotherapy demonstrated promising efficacy in heavily pre-treated HER2-positive MBC (92.5% of the patients with prior trastuzumab and 52.5% with lapatinib), with a median PFS of 7.5 months and an ORR of 50%, which implies that half of the patients could still respond to pyrotinib-based regimen even in later-line settings.

As is well known, efficacy and safety of pyrotinib in patients with HER2-positive MBC have been verified in several pivotal studies. In the phase I study pyrotinib monotherapy demonstrated an ORR of 83.3% in trastuzumab-naive patients and 33.3% in trastuzumab-pre-treated patients [14]. The benefit of continued use of trastuzumab beyond disease progression was also confirmed in earlier studies [8, 9]. The anti-tumor activity and favorable tolerability of dual HER2 blockade with TKI plus trastuzumab has been reported previously and mechanisms of synergistic interaction may involve enhanced apoptosis of cancer cells, increased stabilization and degradation of HER2 receptors, and reversion of resistance to trastuzumab by accumulation of HER2 receptors on the surface of breast cancer cells [20, 21]. Impressively, Murthy and colleagues reported that tucatinib, which was an investigational, oral, highly selective inhibitor of the HER2 tyrosine kinase, was an alternative regimen in heavily pre-treated metastatic breast cancer when combined with trastuzumab and capecitabine, with a median PFS of 7.8 months in the study population and 7.6 months in the brain metastases subgroup [19]. Another single-arm exploratory phase II trial has demonstrated that pyrotinib plus trastuzumab and albumin-bound paclitaxel generated an encouraging pCR rate and an ORR of 100% in the neoadjuvant setting [18]. To our knowledge, there exists no published data investigating the activity and safety of pyrotinib plus trastuzumab and chemotherapy in MBC patients previously treated. Our findings are fairly consistent with and further consolidate the above-mentioned data. The PFS duration reported here is similar with that in the HER2CLIMB trial. Noteworthy, the median PFS of BrM subset that have presumably worse prognosis, was numerically superior to that of the total population. The previous therapeutic lines may predominantly account for this result, since 32.5% of the total population versus 20.0% of BrM subgroup received ≥ 2 lines of therapy previously. Besides, concerning the patients with intracranial metastasis, the PFS duration estimated here (9.4 months) was also longer than that in the HER2CLIMB trial (7.6 months). The potent activity of pyrotinib per se for intracranial metastases may partly account for this finding. Another possible explanation is that local therapy for intracranial metastases (46.7% of the patients with brain metastases received local therapy) improved blood brain barrier permeability and thus the intracranial concentration of anti-tumor drugs increased.

Up to now, evidences on head-to-head comparison of TKI plus trastuzumab versus TKI alone are lacking. A randomized phase II trial, which was designed to assess the efficacy and tolerability of pyrotinib plus capecitabine versus lapatinib plus capecitabine in women with HER2-positive MBC previously treated with taxanes, anthracyclines, and/or trastuzumab, reported that the ORR was 78.5% and the median PFS was 18.1 months in the pyrotinib arm [15]. Similarly, PHENIX study showed that the ORR and median PFS of pyrotinib plus capecitabine were 68.6% and 11.1 months, respectively [17]. Numerically, these findings were superior to the data reported in our study, which might be partly attributed to different distribution of patient characteristics. The phase II study and PHENIX study enrolled patients with no more than 2 lines of prior treatment in the metastatic setting, and nearly 70% of the patients were trastuzumab-naïve. However, 67.5% of the patients in our study received ≥ 2 lines of therapy previously, and almost all patients had received prior trastuzumab for their metastatic disease [15]. Furthermore, our data demonstrated that 52.5% of patients had been exposed to prior lapatinib, which shows a phenomenon that more than half of lapatinib-pre-treated patients still respond to pyrotinib combined with trastuzumab and chemotherapy. Pyrotinib is an irreversible pan-ErbB TKI blocking HER1, HER2, and HER4 while lapatinib is a reversible small dual TKI of HER1 and HER2. Therefore, the response in previous lapatinib-treated patients may partly due to the chemotherapeutic agents and partly due to continuous inhibition of HER2 signal pathway through the reversion of resistance to lapatinib by combination of pyrotinib and trastuzumab. The result further implies the potential activity of pyrotinib plus trastuzumab beyond progression on lapatinib treatment, which merits further evaluation in future studies.

In addition, we further analyzed the predictive factors of PFS and found that liver or/and lung metastases was independent adverse factor for PFS, which suggested that PFS of patients without liver or/and lung metastases were much longer than those with liver or/and lung metastases. It has been reported that live or/and lung is a poor predictor of OS of MBC and a challenge to overcome in clinical practice [23, 24]. In PHENIX trial, subgroup analyses of PFS indicated that pyrotinib plus capecitabine exhibited PFS benefit and significantly decrease recurrence risk compared with placebo plus capecitabine in patients with non-visceral metastases [17]. Wang et al. reported the significant therapeutic effect of pyrotinib on cutaneous metastases of HER2-positive BC for the first time [25]. However, they have not further explored the efficacy and safety of pyrotinib on patients with visceral-metastases. Similarly, data on patients with visceral-metastases were not available in HER2CLIMB trial [19]. Our results demonstrated that patients without liver or/and lung metastases might better benefit from this combination therapy and this finding may provide guidance for patient selection and optimize clinical management.

Referring to safety, the overall incidences of TRAEs were similar to that of previous report and no new TRAEs were reported. The majority of TRAEs were Grade 1 or 2 in severity and most of TRAEs could be managed by dose reduction and supportive treatment. No one discontinued study treatment or died due to TRAEs. Diarrhea, usually occurring on days 4–14 after the first dose, is the most common TRAE which is mainly induced by pyrotinib and could be managed by dose reduction and loperamide, turning mostly tolerable after treatment administered for 1 month. The majority of diarrhea events were Grade 1 or 2, and 25% of them were Grade 3 or 4. Prophylaxis use of loperamide could effectively reduce incidence of diarrhoea caused by neratinib. However, evidence is scarce supporting the prophylaxis use of loperamide in pyrotinib treatment. Of note, no cardiac-related events were recorded in our study. The low incidence of severe adverse events demonstrated the safety of pyrotinib plus trastuzumab in heavily pre-treated MBC patients.

The limitation of this study includes the fact that the study is an initial single-center investigation based on a small Chinese cohort. Another limitation is the relatively low rate of standard care administration in previous lines of treatment among the enrolled patients. As mentioned above, only 6 patients received pertuzumab and trastuzumab as first-line therapeutic regimen and none of them had received TDM-1, ds8201 and tucatinib pre-treatment owing to financial and drug accessibility factors. Therefore, due to the small overall number of patients and the relatively low rate of standard care, this study will always lack sufficient power to observe the efficacy of this combination regimen on patients pre-treated with multiple anti-HER2-target agents as well as the discrepancies between this combination regimen and current standard regimens. However, pyrotinib in combination with trastuzumab and chemotherapy still be a potentially effective alternative regimen in heavily pre-treated patients with HER2-positive MBC. Multi-center randomized controlled trials in larger cohorts are needed to further validate the efficacy and safety of this combination regimen.

In conclusion, pyrotinib in combination with trastuzumab and chemotherapy offer an active option with a favorable safety profile in heavily pre-treated patients with HER2-positive MBC, including those with brain metastases. Multi-center randomized controlled trials are warranted to validate the results.

## Data Availability

The datasets generated during and/or analysed during the current study are available from the corresponding author on reasonable request.
